# Heterologous expression of glutamyl-tRNA reductase gene in *Rhodobacter sphaeroides* O.U.001 to enhance 5-aminolevulinic acid production

**DOI:** 10.1080/13102818.2014.978170

**Published:** 2014-11-07

**Authors:** Gökhan Kars, Ümmühan Alparslan

**Affiliations:** ^a^ Department of Biology, Faculty of Science, Selçuk University, Konya, Turkey

**Keywords:** aminolevulinic acid, biohydrogen, glutamyl-tRNA reductase, heterologous expression, *Rhodobacter sphaeroides* O.U.001

## Abstract

The pathways for synthesis of 5-aminolevulinic acid (5-ALA) use either succinyl-CoA and glycine (C-4 pathway), or glutamate (C-5 pathway). Although *Rhodobacter sphaeroides* synthesizes 5-ALA through the C-4 pathway, it also has the genes coding for the enzymes of the C-5 pathway, except for glutamyl-tRNA reductase. The glutamyl-tRNA reductase gene was cloned from *Rhodospirillum rubrum* and expressed in *R. sphaeroides*; thus, the C-5 pathway was enabled to function upon assembling all the required genes. Consequently, a new and unique bacterial strain producing more 5-ALA was developed. Biohydrogen was also produced in the same bioprocess within a biorefinery approach using sugar beet molasses as substrate. The amount of 5-ALA produced by the modified strain was 25.9 mg/g dry cell weight (DCW), whereas the wild-type strain produced 12.4 mg/g DCW. In addition, the amount of H_2_ generated by the modified and wild-type cells, respectively, was 0.92 L/L culture and 1.05 L/L culture.

## Introduction

Purple non-sulfur (PNS) photosynthetic bacteria such as *Rhodobacter sphaeroides* (*R. sphaeroides*) have the ability to produce a number of value-added products; for instance, 5-aminolevulinic acid (5-ALA), hydrogen, vitamin B12, coenzyme Q10, poly-β-hydroxybutyrate (PHB) and carotenoids. What makes PNS bacteria of particular interest is the possibility to employ them for production of different products in the same bioprocess.

One such value-added product is 5-ALA, which is the precursor of tetrapyrrole porphyrins, vitamin B12 and chlorophyll and has multiple applications in medicine, agriculture and biotechnology. Specifically, it can be used as an anticancer agent, a growth promoter in plants and a biodegradable herbicide and insecticide.[1] However, it does not have widespread usage, since its chemical synthesis is expensive and includes many complex steps.[2] Nevertheless, it is more likely to achieve biosynthesis of 5-ALA at higher quantities in a sustainable bioprocess. A wide range of substrates have been tested for the production of 5-ALA and production of up to 27.5 mmol/L of 5-ALA was achieved with *R. sphaeroides*.[3] In addition to *R. sphaeroides*, several studies have been conducted with green algae [4] and cyanobacteria [5] for the production of 5-ALA in cost-effective bioprocesses. However, the production of 5-ALA needs to be further improved and sustainable and cost-effective bioprocesses need to be developed with the use of renewable substrates instead of pure and synthetic carbon sources.

The pathways for synthesis of 5-ALA use either succinyl-CoA and glycine (C-4 pathway), or glutamate (C-5 pathway).[1] *R. sphaeroides* synthesizes 5-ALA through the C-4 pathway upon condensation of glycine and succinyl CoA by ALA synthetase (ALAS; succinyl-CoA:glycine C-succinyltransferase (decarboxylating); [EC:2.3.1.37]). On the other hand, it also has the first enzyme (glutamyl-tRNA synthetase, RSP_0797, NCBI-GeneID: 3718415, [EC:6.1.1.17]) and the third enzyme (glutamate-1-semialdehyde aminotransferase, RSP_1569, NCBI-GeneID: 3718596, [EC:5.4.3.8]) of the C-5 pathway. However, interestingly, the second enzyme, glutamyl-tRNA reductase, is absent in this bacterium. In the present study, the gene coding for glutamyl-tRNA reductase (the second enzyme) was cloned from *Rhodospirillum rubrum* (*Rsp. Rubrum*, DSM 467, ATCC 11170) and expressed in *R. sphaeroides* O.U.001. In this way, in addition to the C-4 pathway, the C-5 pathway was enabled to function upon assembling all the necessary genes.

Another interesting value-added product that can be produced by PNS bacteria is hydrogen. It can be produced by PNS bacteria under anaerobic conditions in the presence of a light source, using various substrates like organic acids and sugars. Hydrogen is actually the by-product of the enzyme nitrogenase, whose primary function is to reduce molecular nitrogen to ammonium. In the absence of N_2_ and ammonia, however, nitrogenase acts as an adenosine triphosphate (ATP) dependent hydrogenase and all the electrons and ATP molecules are used for hydrogen production.[6] There are many reports on hydrogen production by PNS bacteria in different bioprocesses using various substrates. For instance, *R. sphaeroides* ATCC 17023 can produce 2.31 L H_2_/L culture and 1.62 L H_2_/L culture by using lactate or glucose, respectively.[7] In another study, 13.7 mol H_2_/mol sucrose was obtained by a sequential dark and photofermentation process from molasses.[8] Nevertheless, the processes of biohydrogen production still need to be enhanced with an effective use of feedstock, since most biohydrogen production studies typically use pure and synthetic substrates and the hydrogen production yields are below commercial usage.

In this context, the aim of the present study was to use sugar beet molasses as a renewable and sustainable substrate for the production of biohydrogen and 5-ALA in the same bioprocess. Consequently, a unique and more feasible bioprocess by which considerable amounts of 5-ALA and biohydrogen were produced was obtained using a cost-effective substrate in the context of a biorefinery.

## Materials and methods

### Bacterial strains, plasmids and culture conditions

The bacterial strains and plasmids used in this study are listed in Table 1 and are briefly described below. Wild-type *R. sphaeroides* O.U.001 (DSM 5864, DSMZ GmbH, Germany) was used for the production of 5-ALA and biohydrogen. The second gene (glutamyl-tRNA reductase, Rru_A0749, EC:1.2.1.70) in the C-5 pathway was taken from *Rsp. rubrum* (DSM 467, ATCC 11170, DSMZ GmbH, Germany) and expressed in *R. sphaeroides*. Both bacteria were maintained according to the supplier's recommendation (*Rhodospirillaceae* medium No:27). *Escherichia coli* XL1 Blue (Stratagene) was used as a general plasmid host. *E. coli* S17-1 (λ pir) is a special strain used as plasmid donor in conjugation or diparental mating.[9] Here, it was used to deliver the construct to *R. sphaeroides* by conjugation. pBBR1MCS2 [10] was used for the cloning and heterologous expression of the glutamyl-tRNA reductase gene in *R. sphaeroides*. *E. coli* strains were maintained in Luria–Bertani (LB) medium supplied with antibiotics in the following concentrations: kanamycin (25 μg/mL) and tetracycline (10 μg/mL).

**Table 1.** Plasmids and bacterial strains used in this study.

Strains/plasmids	Characteristics/genotype	Reference
Strains		
*E. coli* XL1 Blue	Δ(*mcrA*)183, Δ(*mcrCB-hsdSMR-mrr*) 173, *endA1*, *supE44*, *thi-1*, *recA1*, *gyrA96*, *relA1 lac* (F′ *proAB lacIqZ*ΔM15 Tn10 (Tet^r^))	Stratagene
*E. coli* S17-1	294 (*recA pro res mod*) Tp^r^, Sm^r^ (pRP4-2-Tc::Mu-Km::Tn7), λ pir	Simon et al. [9]
*R. sphaeroides* O.U 001	Wild type	DSM 5864
*Rsp. rubrum*	Wild type	DSM 467
		
Plasmids		
pBBR1MCS2	Km^r^, *pBBR1 replicon*, *mob+*	Kovach et al. [10]
pALA3	Glutamyl-tRNA reductase gene cloned into pBBR1MCS2 vector	This work

In order to develop a cost-effective process, sugar beet molasses was supplied from a sugar factory (Konya Şeker, Turkey) and used as substrate. It is the viscous dark brown by-product of the refining of sugar beets and contains 50% (w/w) sugar, mainly sucrose. Sugar-containing medium with sucrose concentration of 28 g/L was used based on our previous results [11] that in this medium, 5-ALA (35 mg/g dry cell weight (DCW)) and H_2_ (1.01 L/L culture) can be obtained using molasses. Fifty-five milliliter glass bioreactors with a final culture volume of 50 mL were used. After 10% inoculation, the anaerobic cultures made by flushing the bioreactors with argon for 3 min were incubated at 29 °C without shaking under 200 Watt/m^2^ illumination provided by 100 W incandescent light bulbs.

### Cloning and expression of the glutamyl-tRNA reductase gene

The enzymes used, including kinase, phosphatase, ligase, DNA polymerases and restriction endonucleases, were supplied from Thermo Scientific Inc. (Germany).

The glutamyl-tRNA reductase gene was obtained by polymerase chain reaction (PCR) in a thermal cycler (Boeco, Germany) using the genomic DNA of *Rsp. rubrum* as template. The 1811 bp long DNA fragment including both the glutamyl-tRNA reductase gene and its upstream genetic elements was synthesized using designed primers (forward primer: 5′-GAATTCGTCACCACCGATCT-3′; reverse primer: 5′-GGCTCAGGTTCTCTTCCAAA-3′). The PCR programme was as follows: 30 s at 98 °C for pre-denaturation, 35 cycles of amplification step (10 s at 98 °C, 30 s at 55 °C and 45 s at 72 °C) followed by a final extension at 72 °C for 5 min. The reaction was performed in the presence of 3%, 5%, 7%, 9% or 11% (w/v) dimethyl sulphoxide (DMSO), using a high-fidelity DNA polymerase enzyme (Phusion, Thermo Scientific) in a total volume of 20 μL.

PCR products (250 μL) were precipitated with 3 mol/L sodium acetate (pH 5.2) and phosphorylated with T4 polynucleotide kinase, according to the manufacturer's protocol. The mixtures were then loaded into an agarose gel (1%) and purified using a gel extraction kit (Qiagen). The PCR products became ready to ligate into the vector. pBBR1MCS2, which can replicate in *R. sphaeroides*, was used to clone and express the glutamyl-tRNA reductase gene. The vector was cut with *Eco*RV, dephosphorylated by calf intestinal alkaline phosphatase (CIAP) to prevent self-ligation and purified with a gel extraction kit (Qiagen). The insert and vector were mixed in a 3:1 molar ratio to ensure successful ligation in the presence of T4 DNA ligase in a total volume of 20 μL. After 1-h incubation at 22 °C, a few microliters of ligation mixture were transformed to *E. coli* XL1Blue through CaCl_2_-mediated chemical transformation.

After transformation into *E. coli* XL1Blue, several white colonies were investigated to find the correct recombinant clone. For this purpose, plasmid isolations were done from these colonies and the plasmids were cut with *Hinc*II to confirm the cloning and find the orientation of the insert. Upon confirmation of the cloning, the correct recombinant vector carrying the glutamyl-tRNA reductase gene was denoted as pALA3.

For the delivery of the construct into *R. sphaeroides*, diparental mating (conjugation) was applied. The construct was first transferred into *E. coli* S17-1 (λpir) strain, which provides the transfer function by a *tra* gene on the chromosome. The *mob* region required for the transfer was provided by the construct. Fifty-milliliter cultures of *E. coli* S17.1 (λpir) containing the construct and 50 mL of wild-type *R. sphaeroides* O.U.001 were pelleted by centrifugation. The pellets were re-suspended and mixed in 5 mL of Biebl and Pfenning minimal medium.[12] The cell mixture was re-pelleted and re-suspended in 1 mL of minimal medium and spotted onto a 0.45 μm pore size nitrocellulose filter that was previously placed on an LB plate without antibiotic. After 6-h incubation at 30 °C, the cell mixture was washed from the filter paper with 1 mL of minimal medium and collected into a tube. Then, several microliters of this mixture were spread onto selective minimal medium with kanamycin. Since the *E. coli* S17-1 (λpir) strain is a proline auxotroph, it was not able to form colonies on minimal medium but *R. sphaeroides* colonies containing the construct appeared on the selective medium within 2–3 days. *R. sphaeroides* colonies grown on the selective media with kanamycin were further cultured and plasmid isolation was performed from these cells to confirm the maintenance of pALA3 in *R. sphaeroides*. The vector isolated from *R. sphaeroides* was cut with *Kpn*I and used as template in PCR to amplify the glutamyl-tRNA reductase gene. This proved that pALA3 was successfully delivered to *R. sphaeroides* by conjugation and it was stably maintained in the bacterium.

The transcription of the glutamyl-tRNA reductase gene in *R. sphaeroides* was investigated by reverse transcription PCR (RT-PCR). First, total RNA was isolated with TRI Reagent (Sigma), according to the manufacturer's instructions, using 4 mL of overnight *R. sphaeroides* culture containing pALA3. Then, c-DNA synthesis and subsequent PCR were done with specifically designed primers (GTR-1 forward: 5′-GCGTGGAGATCTTTGGTCAT-3′, GTR-1 reverse: 5′-TTGACCTGCCCCAAAATATG-3′ and product size: 211 bp). PCR was performed using 1 μL of cDNA with *Taq* DNA polymerase in a total volume of 25 μL. The PCR programme was as follows: 5 min at 95 °C for pre-denaturation, 30 cycles of amplification (30 s at 95 °C, 30 s at 51.5 °C and 30 s at 72 °C), followed by a final extension step at 72 °C for 5 min. Finally, PCR products were visualized by agarose gel (1%) electrophoresis.

### Experimental design for biohydrogen and 5-ALA production

The experiments were done by using a metabolically engineered strain and a wild-type strain which was transformed with bare vector (pBBR1MCS2) as a control. Since molasses is a viscous and dark brown by-product, it was first diluted with distilled water to prepare the medium with 28 g/L of sucrose. In addition to 2 mmol/L of glutamate added as a nitrogen source, K_2_HPO_4_, MgSO_4_·7H_2_O, CaCl_2_·2H_2_O, FeSO_4_, MoO_4_·2H_2_O and vitamin solution (Thiamine, Niacin, Biotin) were included in the culture medium as described earlier.[11] After 10% inoculation, the anaerobic cultures were incubated under the conditions defined above. During the bioprocess (circa the 90th hour), levulinic acid (1.74 g/L, 15 mmol/L) was added into the cultures to enhance 5-ALA production, as recommended by Choi et al. [13]

The cell density of the cultures was monitored by measuring the absorbance of the cultures at 660 nm at specific time intervals. DCW was also calculated based on preliminary optical density (OD) experiments showing that one unit OD_660_ of *R. sphaeroides* O.U. 001 is equivalent to dry weight of 0.421 g/L. The pH changes in the cultures were also monitored and recorded. The evolved gas was collected in water-filled graduated tubes during the bioprocess and then the purity was determined at the end of the bioprocesses by gas chromatography (Shimadzu GC-2010 Plus, Japan) as described previously.[11] The amount of hydrogen accumulation was given as the liters of hydrogen produced per liter of culture (L H_2_/L culture) and as the yield (mol H_2_/mol sucrose), which was calculated by taking the temperature to be 29 ºC and the pressure to be 101.3 kPa (i.e. 1 atm). After the completion of the batch processes, the cell-free culture media were lyophilized and re-suspended in 2 mL of distilled water. The amount of 5-ALA in this suspension was determined spectrophotometrically using a standard curve.[14]

### Statistical analysis

Hydrogen production experiments were independently repeated two times. Two-tailed *t*-test was used to determine the significant differences between mean values (*p* < 0.05). Each value in the graphs is the mean from two replicates (±standard deviation).

## Results and discussion

### Cloning and heterologous expression of the glutamyl-tRNA reductase gene

**Figure 1.**
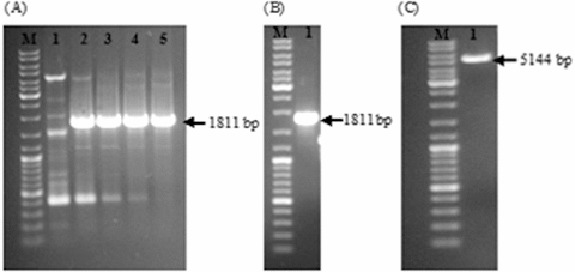
Optimization of PCR amplification of the glutamyl-tRNA reductase gene by using various DMSO concentrations (A); Lane 1: 3% (w/v); Lane 2: 5% (w/v); Lane 3: 7% (w/v); Lane 4: 9% (w/v); and Lane 5: 11% (w/v). PCR product after tailoring the ends and purification by gel extraction kit (B). *EcoR*V cut, dephosphorylated and purified pBBR1MCS2 (C). DNA ladder (SM0331 Fermentas, M) was loaded into the first well.

The 1811-bp long DNA fragment including both the glutamyl-tRNA reductase gene and its upstream genetic elements was successfully amplified by PCR (Figure 1(A)). After tailoring the ends of the PCR product and subsequent purification by gel extraction kit, the amplified fragment became ready for ligation (Figure 1(B)). Similarly, pBBR1MCS2 was cut with *Eco*RV, CIAP treated to prevent self-ligation and purified for ligation (Figure 1(C)). Finally, the amplified DNA fragment was blunt-end cloned into pBBR1MCS2.

**Figure 2.**
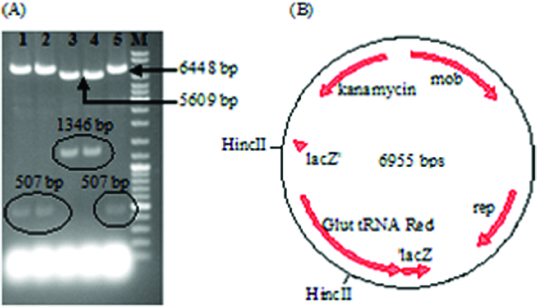
Selection of the correct recombinant vector by *Hinc*II digestion (A); Lanes 1–5: *Hinc*II digestion of five putative recombinant vectors isolated from five different colonies. Only the third and the fourth vector gave the expected bands. DNA ladder (SM0331 Fermentas, M) was loaded into the last well. Illustration of the correct recombinant vector designated as pALA3 (B).

Following the transformation of *E. coli* XL1Blue, five white colonies were selected and investigated to determine if they were correct recombinant colonies. In this context, the plasmids were isolated from these colonies and they were cut with *Hinc*II. Two of the tested plasmids gave the expected DNA fragments (5609 and 1346 bp) and they were designated as pALA3 (Figure 2(A)). In these two constructs, the transcriptional orientation of the glutamyl-tRNA reductase gene was the same as that of *LacZ* and the kanamycin resistance gene (Figure 2(B)), avoiding the possibility for the transcription of one gene to decrease the transcription frequency of the neighboring one.

**Figure 3.**
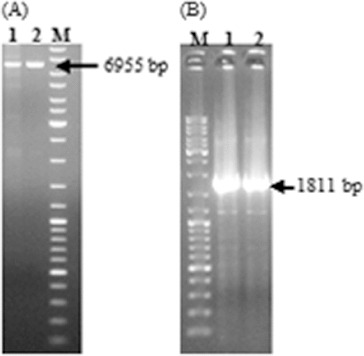
Confirmation of the replication of pALA3 in *R. sphaeroides* by *Kpn*I digestion (A); Lane 1: digestion of the vector isolated from *R. sphaeroides* and Lane 2: digestion of pALA3 as control. Confirmation of the presence of glutamyl-tRNA reductase gene in the isolated vector, using the plasmid as template in PCR (B); Lane 1: PCR product obtained by using vector isolated from *R. sphaeroides* and Lane 2: PCR product obtained by using pALA3 as control. DNA ladder (SM0331 Fermentas) was designated as M.

**Figure 4.**
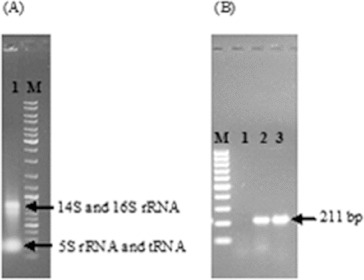
Total RNA isolation from *R. sphaeroides* transformed with pALA3 (A). Expression analysis of glutamyl-tRNA reductase gene by RT-PCR (B); Lane 1: RT^−^ (without reverse transcriptase); Lane 2: RT^+^ (with reverse transcriptase); and Lane 3: positive control (with pALA3). DNA ladder (100 bp) was loaded (M).

After the successful construction of the vector, it was first transferred to *E. coli* S17-1 (λpir) to mobilize the construct into *R. sphaeroides* by conjugation. Then, wild-type *R. sphaeroides* O.U.001 and *E. coli* S17.1 (λpir) containing the construct were mated and *R. sphaeroides* colonies transformed with pALA3 were selected on minimal medium with kanamycin. In order to ensure the maintenance and replication of the vector in *R. sphaeroides*, plasmid isolation was performed from these cells and they were cut with *Kpn*I. The vector was linearized and the expected 6955 bp band was obtained (Figure 3(A)). Next, the presence of the glutamyl-tRNA reductase gene in the vector was confirmed by PCR using these plasmids as a template and the expected 1811 bp DNA fragment was successfully amplified (Figure 3(B)). Subsequent to the confirmation that pALA3 was successfully delivered to *R. sphaeroides* by conjugation and that it was stably maintained in the bacterial cells, the transcription of glutamyl-tRNA reductase gene was investigated by RT-PCR. In this context, total RNA isolation was first carried out using anaerobically grown *R. sphaeroides* containing pALA3 as shown in Figure 4(A). 16S and 14S rRNA, which arise from the cleavage of 23S rRNA,[15] were observed together with 5S rRNA and tRNA, without any contaminating DNA in the agarose gel. Following the cDNA synthesis, PCR was performed and the result is illustrated in Figure 4(B). Thus, the transcription of the glutamyl-tRNA reductase gene in *R. sphaeroides* was demonstrated. It is known that transcription does not always guarantee successful translation or functional enzyme synthesis. For this reason, the effect of heterologous gene expression on the production of 5-ALA was investigated by comparing the amount of 5-ALA produced by the wild-type and the modified strain.

### Production of biohydrogen and 5-ALA

**Figure 5.**
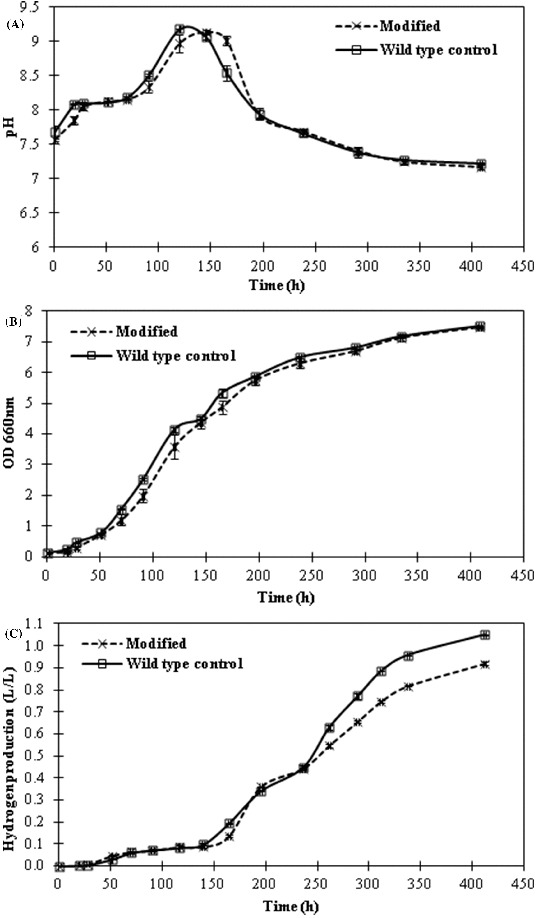
pH values (A); optical density (OD) measured at 660 nm (B); and hydrogen production (C) during the growth of wild-type and modified *R. sphaeroides* strain. Values are means from two replicates (±standard deviation).

5-ALA and biohydrogen production studies were carried out with both the metabolically engineered strain and the wild-type *R. sphaeroides* which was transferred with a bare vector as a control. The pH of the medium was initially buffered to 6.8 and was not controlled during the process. The pH changes of the cultures are illustrated in Figure 5(A). Both the modified and the wild-type strain followed almost the same pH pattern. Initially, the pH increased up to about 9.2 between 120 and 145 h and then returned back to about 7.3. This rise in pH to about 9.2 did not have any negative effects on the growth and hydrogen production of *R. sphaeroides* because substantial amounts of growth and hydrogen production were observed after this point of the cultivation process (Figure 5(B) and (C)). Similar pH changes were observed in our previous studies where different concentrations of sugar were tested using molasses.[11] As a whole, consistent results were obtained in both studies with slight variations. Likewise, the growth of wild-type and modified *R. sphaeroides* were monitored by measuring the absorbance at 660 nm (Figure 5(B)). Both the wild-type and modified strains reached almost the same high cell densities (OD_660_ of 7.94 and DCW of 3.34 g/L). This result affirms that sugar beet molasses supports the growth of photosynthetic bacterium *R. sphaeroides* significantly, which is probably due to the rich composition of molasses. The defined media with organic acid also sustain the growth of *R. sphaeroides* well but at relatively lower cell densities, e.g. OD_660_ was 1.9 (DCW of 0.8 g/L) and 3.4 (DCW of 1.43 g/L), using malate and acetate, respectively.[16,17] The production of a considerable amount of PHB (70.4%, w/w) by *R. sphaeroides* was reported with the use of sugar refinery wastewater.[18] Based on these data, sugar beet molasses can be considered to serve as an excellent carbon, electron and energy source for various metabolic activities.

The gas chromatography results about the obtained amounts of pure hydrogen are illustrated in Figure 5(C). While 1.05 L H_2_/L culture (0.59 mol H_2_/mol sucrose) was produced by the wild-type *R. sphaeroides*, 0.92 L H_2_/L culture (0.50 mol H_2_/mol sucrose) was produced by the modified strain. These results indicate that more energy and reducing equivalents were directed towards the nitrogenase enzyme and thus, a little more hydrogen accumulation was achieved with the wild-type cells. On the other hand, the modified cells allocated much of their energy and reducing equivalents to the 5-ALA production pathways and therefore relatively less hydrogen was obtained. These results are in agreement with our previous findings that 0.5 mol H_2_/mol sucrose was obtained under similar conditions,[11] which suggests that heterologous expression of glutamyl-tRNA reductase gene in *R. sphaeroides* did not drastically lessen the amount of hydrogen production. There are, however, also reports for relatively higher amounts of hydrogen obtained in different bioprocesses. For example, 3.47 mol H_2_/mol sucrose was produced in a dark fermentation process using a mixed anaerobic culture.[19] An even higher yield (13.7 mol H_2_/mol sucrose) was obtained in a sequential dark and photofermentation process with a mutant strain of *R. capsulatus*.[8]

In this study, we also aimed at improved production of 5-ALA by introducing a second 5-ALA production pathway (C-5 pathway) in addition to the existing C-4 pathway. In this context, the missing glutamyl-tRNA reductase gene was successfully expressed in *R. sphaeroides*. The concentrations of 5-ALA produced by the modified and the wild-type strain were 25.9 and 12.4 mg/g DCW, respectively. Thus, it was shown that *R. sphaeroides* produced more 5-ALA upon establishing a second 5-ALA production pathway. A wide range of 5-ALA quantities have been reported. For instance, 0.45–226 μg of 5-ALA/g DCW were obtained in different halotolerant strains of *R. sphaeroides*.[20] In comparison with these, significant amounts of 5-ALA were produced with both modified and wild-type strains in this study. On the other hand, it was thought that the presence of antibiotic and vector exerted a negative effect on growth, hydrogen and 5-ALA production. For example, when comparing with the results from our previous study [11] where antibiotic was not used, in this study either a delay or a decrease in cell density, hydrogen and 5-ALA productions occurred. For this reason, in the future, the glutamyl-tRNA reductase gene should be integrated into the chromosome to eliminate vector usage and the accompanying negative effects. Further studies for improvements in the gene expression system are underway.

## Conclusions

In this study, a second 5-ALA production pathway (C-5 pathway), in addition to the C-4 pathway, was enabled by heterologous expression of the missing glutamyl-tRNA reductase gene in *R. sphaeroides*. Thus, a new and unique bacterial strain producing more 5-ALA was developed by implementing a novel idea. The gene expression system could be optimized in the future or the glutamyl-tRNA reductase gene could be integrated into the chromosome to eliminate vector usage and the accompanying negative effects. Biohydrogen was also produced in the same bioprocess within a biorefinery approach using sugar beet molasses as substrate. As a result, a unique and more feasible bioprocess was obtained.
